# Partial Oxidation Strategy to Synthesize WS_2_/WO_3_ Heterostructure with Enhanced Adsorption Performance for Organic Dyes: Synthesis, Modelling, and Mechanism

**DOI:** 10.3390/nano10020278

**Published:** 2020-02-06

**Authors:** Guiping Li, Yongli Wang, Jingtao Bi, Xin Huang, Yafei Mao, Liang Luo, Hongxun Hao

**Affiliations:** 1 National Engineering Research Center for Industry Crystallization Technology, School of Chemical Engineering and Technology, Tianjin University, Tianjin 300072, China; ligp@tju.edu.cn (G.L.); jingtaob@gmail.com (J.B.); x_huang@tju.edu.cn (X.H.); maoyafei@tju.edu.cn (Y.M.); 2016207086@tju.edu.cn (L.L.); 2 Co-Innovation Center of Chemical Science and Engineering, Tianjin 300072, China

**Keywords:** WS_2_/WO_3_ heterostructure, surface property, partial oxidation, hydrophilicity, adsorption mechanism

## Abstract

In this work, a facile oxidation strategy was developed to prepare novel tungsten disulfide/tungsten trioxide (WS_2_/WO_3_) heterostructures for adsorbing organic dyes efficiently by combining the hydrophilic property of WO_3_ and the superior dye affinity of WS_2_. The structural and elemental properties of the synthesized hybrid materials were systematically investigated, and the results demonstrated the retained flower-like morphology of the primitive WS_2_ and the successful introduction of WO_3_. Furthermore, surface properties such as a superior hydrophilicity and negative-charged potential were also demonstrated by a water contact angle characterization combined with a Zeta potential analysis. The performance of the obtained WS_2_/WO_3_ hybrid materials for removing Rhodamine B (RhB) from wastewater was evaluated. The results showed that the maximum adsorption capacity of the newly synthesized material could reach 237.1 mg/g. Besides, the adsorption isotherms were also simulated by a statistical physics monolayer model, which revealed the non-horizontal orientation of adsorbates and endothermic physical interaction. Finally, the adsorption mechanism and the recyclability revealed that the partial oxidation strategy could contribute to a higher adsorption capacity by modulating the surface properties and could be applied as a highly efficient strategy to design other transition metal dichalcogenides (TMDs) heterostructures for removing organic dyes from wastewater.

## 1. Introduction

In the past several decades, it was estimated that millions of tons of dyes were produced annually, and amount of dyes were discarded into water as waste [1,2,3]. These organic dyes, which are toxic, non-biodegradable, and even carcinogenic, had posed serious threats to human health and marine organisms [4]. Consequently, it is urgent to develop low-cost and efficient methods to remove these organic dyes from wastewater. Various technologies have been developed and employed for treating wastewater, including membrane filtration, electrochemical decolorization, photocatalytic degradation, chemical oxidation/reduction, biodegradation, and adsorption [2,5,6,7,8,9,10,11,12,13,14,15]. Among them, adsorption is considered a promising candidate to eliminate dyes from industrial wastewater due to the low capital cost, simplicity of operation, and low introduction of toxic byproducts. A large number of materials have been developed and utilized in adsorption, such as carbon nanotubes, metal-organic framework (MOF), and graphene, which have the advantages of a high surface area, hydrophilic properties, and intense interactions between adsorbents and adsorbates [4,16,17,18,19,20,21].

Recently, materials with a layered structure started to be investigated and applied in the field of adsorption. They have already shown unique chemical and physical advantages compared with the bulk counterparts since the layered structures possess open channels to provide more active sites for adsorbates. Among these materials, the transition metal dichalcogenides (TMDs) are attractive due to their sandwich structures where covalently bound (X–M–X) trilayers are held together by van der Waals forces, which are conducive to forming a layered structure [22,23]. More importantly, strong polar X–S covalent bonds located at lamellar layers’ edges could attract polar species like organic dyes [22,24]. However, the layer nanosheets are inevitably restacked and agglomerated owing to their high surface energy, which would result in the decrement of adsorption sites and the corresponding adsorption effect [25]. Therefore, some studies were conducted to assemble 2D nanosheets in hierarchical three-dimensional (3D) structures to prevent their restacking or agglomeration. For example, 3D flower-like MoS_2_ synthesized by Geng et al. exhibited a maximum adsorption capacity of 49.2 mg/g for RhB dye [26]. Furthermore, He et al. introduced porosity into the flower-like MoS_2_ adsorbents, resulting in an increment of the adsorption capacity for RhB (163 mg/g), with an extension of the adsorption duration [27]. These reported investigations mainly concentrated on MoS_2_ whereas other TMD materials are still less explored. Moreover, most reported studies were primarily focused on the construction of different 3D structures, while investigations on the surficial modification are limited.

In this study, WS_2_, a typical TMD material with a similar structure to MoS_2_ was selected as the model material to study the relationship between surficial modification and adsorption behavior. Herein, *N*-methy-l-2-pyrrolidone (NMP) was applied as the solvent medium to enhance the vulcanization of WCl_6_, and a partial oxidation method was introduced to modulate the surficial conditions of WS_2_ to synthesize WS_2_/WO_3_ hybrid materials. Besides, characterizing methods including scanning electron microscopy (SEM), transmission electron microscopy (TEM), X-ray diffraction (XRD), X-ray photoelectron spectroscopy (XPS), Fourier transform infrared (FTIR) spectra, Zeta potential, and contact angle measurements were utilized to confirm the successful fabrication of WS_2_/WO_3_ heterostructures and to explore the properties of surface charges and hydrophilicity after partial oxidation. Furthermore, batch absorption experiments were systematically conducted, and a synergistic effect between the hydrophilic property of WO_3_ and superior dye affinity of WS_2_ was observed. Finally, the adsorption kinetics and isotherms were investigated to better understand the adsorption process, and the adsorption mechanism was revealed.

## 2. Materials and Methods

### 2.1. Materials

Tungsten hexachloride (WCl_6_, ≥99 wt% purity), N-methyl-2-pyrrolidone (NMP, ≥99.5% purity), sublimed sulfur (S, 99.5 wt% purity), and Rhodamine B (99 wt% purity) were purchased from Aladdin Co., Ltd. (Shanghai, China). NaOH (96.0 wt% purity) and HNO_3_ (68 wt% purity) were purchased from Tianjin Kermel Co., Ltd. (Tianjin, China). All reagents were used as received without further purification, and ultra-pure water was prepared in our laboratory and used throughout.

### 2.2. Synthesis of WS_2_, WS_2_/WO_3_ Heterostructures and WO_3_

**Figure 1:**
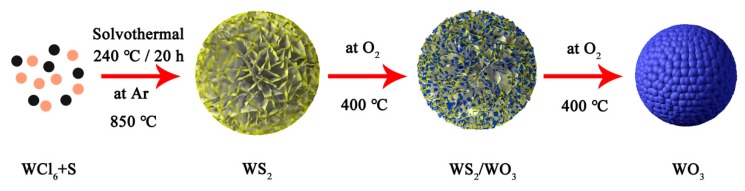
Schematic illustration of the synthetic procedure of WS_2_, WS_2_/WO_3_, and WO_3_.

The preparation procedures of WS_2_, WS_2_/WO_3_ hybrids, and WO_3_ are illustrated in Figure 1. The WS_2_ microflowers were first synthesized by using a solvothermal method [28]. In general, WCl_6_ (0.894 g) was dissolved in NMP (70 mL), and this suspension was stirred for 10 min to obtain a dark brown solution. Next, sublimed sulfur was added into the mixture accompanied with an ultrasonic treatment for 20 min until a homogenous solution was formed. Subsequently, the solution was transferred into a 100 mL PolyPhenyLene-lined stainless steel autoclave and was heated at 240 °C for 20 h. After the solvothermal process, the black precipitates were collected and dried under vacuum at 100 °C for 12 h. The black powder was WS_2_ and was thereafter annealed at 850 °C for 2 h under argon atmosphere to increase the crystallinity. 

After the synthesis of WS_2_, the solids were treated in a furnace at 400 °C for *n* (*n* = 5, 30, and 60) min under oxygen flow (100 mL/min) to obtain a series of partially oxidized products which were denoted as WSO-n. Finally, pure WO_3_ was obtained with an extending duration of 4 h.

### 2.3. Material Characterizations

X-ray diffraction patterns of the obtained materials were recorded by an X-ray powder diffractometer (D/max 2500, Rigaku, Tokyo, Japan) with Cu Kα radiation (λ = 1.5418 Å). A thermal gravimetric analysis (TGA) was performed under oxygen flow on a NETZSCH TG 209F3 analyzer (Bavaria, Germany). Morphological information was recorded by scanning electron microscopy (Nanosem 430, FEI, Eindhoven, Netherlands) and transmission electron microscopy (Tecnai G^2^ F20, FEI, Eindhoven, Netherlands). Elements contents and their chemical states were analyzed by X-ray photoelectron spectroscopy (ESCALAB 250XI, Al Kα, Thermo, MA, USA) which was calibrated by contaminant carbon at 284.8 eV. Zeta potentials were analyzed by Malvern Zetasizer Nano Series (Nano ZSP, Malvern, Worcestershire, UK) in the pH range of 10 to 2. The Fourier transform infrared (FTIR) spectra with the wavelength range of 4000–400 cm^−1^ were obtained using a Bruker Alpha spectrometer (Regensburg, Germany). Water contact angles were measured on a Dataphysics OCA contact angle meter.

### 2.4. Batch Adsorption Experiments

All the following adsorption experiments except the adsorption isotherms were conducted at 30 °C. The kinetic studies of different obtained materials toward the absorption of RhB were investigated via the following procedure: 20 mg of adsorbents were put into a 40 mL RhB solution (200 mg/L), after which the mixture was placed in a dark place and stirred vigorously. The mixture was sampled at predetermined time intervals, and then the solid absorbent was separated from the solution through centrifugation. The concentration of the remaining RhB dye on solution was measured via a UV–vis spectrophotometer (Hitachi U4100 spectrophotometer, Kyoto, Japan), at the maximum adsorption wavelength of RhB (554 nm). The adsorption amount $Q_{t}$ (mg/g) of RhB was expressed as follows:

(1) $$Q_{t}=\frac{(C_{0}-C_{t})V}{m},$$

where $C_{0}$ and $C_{t}$ (mg/L) represent the concentrations of remaining RhB at times 0 and t (min), respectively. V (L) is the volume of RhB solution, and m (g) is the mass of the adsorbent. 

The adsorption isotherms of RhB on WSO-5 were conducted at 30, 40, and 50 °C with a solution pH of 6. Specifically, the adsorption experiments were performed by putting 20 mg of the as-prepared materials into a 40 mL RhB solution with different concentrations (100–400 mg/L) under a constant stirring rate of 200 rpm. After adsorption for 5 h to reach the equilibrium condition, the solid phase was separated from the liquid phase by centrifugation, and the equilibrium concentration of RhB was measured. The relative adsorption capacity of RhB was calculated by the following formula:

(2) $$Q_{e}=\frac{(C_{0}-C_{e})V}{m},$$

where $Q_{e}$ (mg/g) represents the equilibrium adsorption capacity of RhB, and $C_{e}$ (mg/L) is the concentration of RhB at equilibrium status.

The effect of the pH was explored by adding 20 mg adsorbent into a 40 mL RhB solution (200 mg/L) at a series of pH levels (10 to 2). The required pH values were adjusted by adding a negligible volume of 0.1 M HNO_3_ and NaOH solutions. Furthermore, the effect of the adsorbent dosage was also studied by conducting a 40 mL RhB solution (200 mg/L) with a varying mass of WSO-5 from 10 mg to 60 mg for 5 h. The concentration of the remaining RhB in the solution was measured with the aforementioned method.

## 3. Results and Discussion

### 3.1. Characterizations

**Figure 2:**
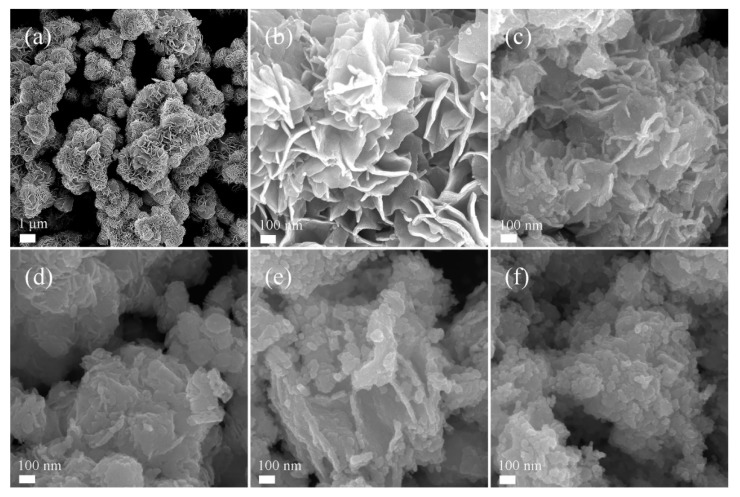
The morphologies of WS_2_, WS_2_/WO_3_, and WO_3_. (**a**,**b**) SEM images of WS_2_; (**c**–**e**) SEM images of WSO-5, WSO-30, and WSO-60, respectively; (**f**) SEM image of WO_3_.

**Figure 3:**
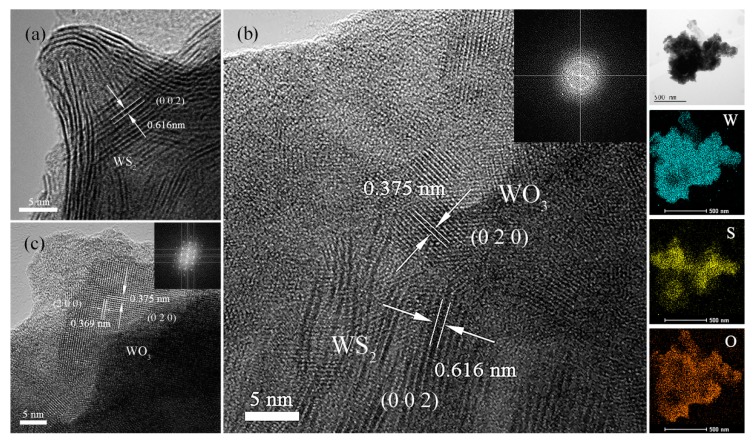
High-resolution TEM (HRTEM) images of (**a**) WS_2_, (**b**) WSO-5, (**c**) WO_3_, and (**b** inset) elemental mapping of WSO-5.

The microstructures and morphologies of WS_2_, WSO-5, WSO-30, WSO-60, and WO_3_ were characterized by SEM and TEM, and the results are shown in Figure 2 and Figure 3. The SEM images in Figure 2a,b demonstrate that the synthesized WS_2_ has a uniform 3D flower-like morphology, which consists of intercrossed nanoflakes with a thickness of several nanometers. According to the thermal-gravimetric analysis (TGA) result (Figure S1), WS_2_ starts to lose weight at about 360 °C. Thus, WS_2_ microflowers were determined to be calcined at 400 °C for the partial oxidation of WS_2_ to WO_3_. The obtained WS_2_/WO_3_ heterostructures were also characterized by SEM (Figure 2c–e). It can be clearly seen that the well-defined flower-like structures crumble gradually with an increase in the calcination time. As displayed in Figure 2c,d, the flower-like microstructures in WSO-5 and WSO-30 are retained, while there is almost no flake morphology in WSO-60. Finally, the three-dimensional structures were entirely destroyed into aggregating particles in WO_3_ after the thoroughly oxidation of WS_2_ into WO_3_ (Figure 2f). 

The microscopic structures of the obtained composites were further investigated using high-resolution transmission electron microscopy (HRTEM), and the results are displayed in Figure 3. Initially, the interlayer spacing of WS_2_ nanosheets displayed in the Figure 3a, measured to be 0.616 nm, can be ascribed to the (0 0 2) plane of WS_2_ [28]. For the sample with a short partial oxidation time of 5 min, a new lattice fringe with *d* = 0.375 nm appears beside the interlayer spacing of 0.616 nm according to Figure 3b, ascribed to the characteristic interplane distance of the (020) crystallographic plane of WO_3_. The elemental mapping results of WSO-5 further confirm the uniform distribution of W, S, and O elements on the nanoflakes, suggesting that the WS_2_ and WO_3_ species are interconnected on the nanoscale rather than isolated from each other (Figure 3b inset). These polycrystalline structures are also confirmed by a Fast Fourier Transform (FFT) pattern in Figure 3b. Finally, the lattice fringes of WS_2_ disappear, and only the lattice fringes of WO_3_ remain (Figure 3c) when the composites are entirely transformed into WO_3_ particles.

**Figure 4:**
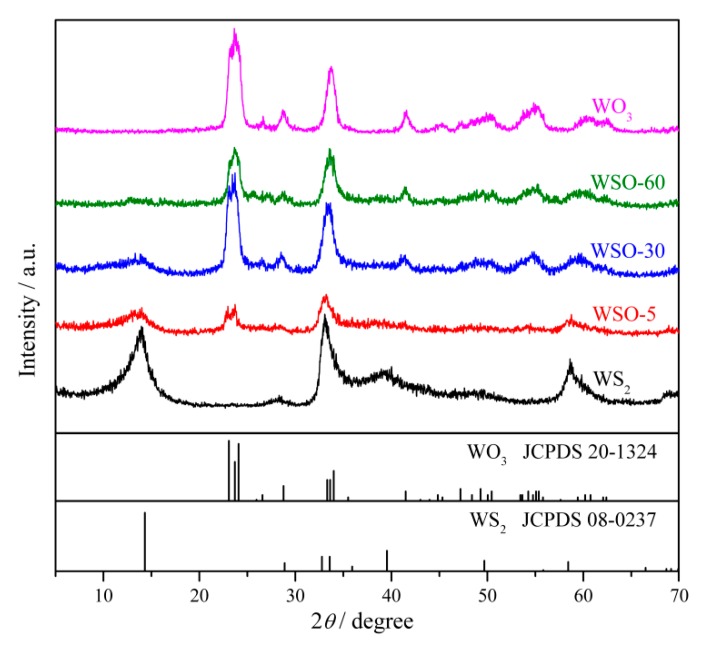
The PXRD patterns of different materials WS_2_/WO_3_, WS_2_, and WO_3_.

The crystalline information is demonstrated by XRD patterns (Figure 4). From the pattern of pure WS_2_, several broad peaks at 14.5°, 32.7°, 33.6°, 39.5°, and 58.4° are in good agreement with the (002), (100), (101), (103), and (110) planes of the hexagonal phase of WS_2_ (JCPDS Card No.08-0237) [29]. With the increase of the calcining time, the intensity of the (0 0 2) peak decreases gradually from WSO-5 to WOS-60. Simultaneously, the peaks at about 24.0° and 33.6°, which are ascribed to the (0 2 0) and (2 2 0) peaks of WO_3_, emerge and get strengthened. This phenomenon also suggests the successful construction of WS_2_/WO_3_ heterostructures. Moreover, pure WO_3_ was also analyzed, and it was found that WO_3_ possesses characteristic peaks at 23.1°, 23.8°, 24.1°, 33.6°, and 34.0°, corresponding to the crystal facets (001), (020), (200), (201), and (220) of WO_3_ (JCPDS Card No.20-1324) respectively [30].

**Figure 5:**
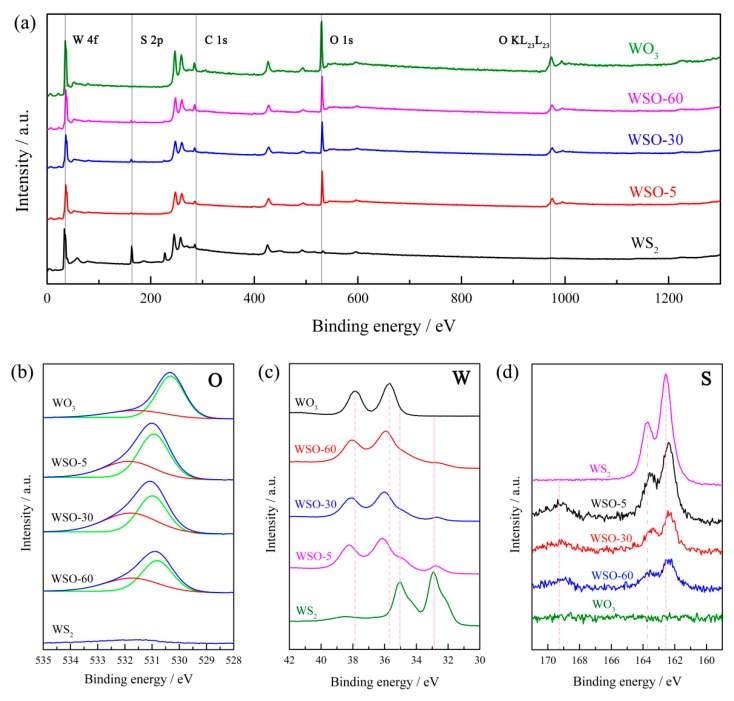
XPS spectra for WS_2_, WS_2_/WO_3_, and WO_3_. (**a**) Full-range XPS spectra, (**b**) O 1s spectra, (**c**) W 4f spectra, and (**d**) S 2p spectra.

For the purpose of examining the chemical compositions and electronic states of elements of WSO-n, X-ray photoelectron spectroscopy (XPS) was further utilized in this work. It can be seen from the XPS full-range spectra (Figure 5a) that these obtained composites exhibit characteristic peaks of C, W, O, and S elements. The characteristic peak of C 1s situated at ~284.8 eV, ascribed to the contaminant carbon, was utilized to calibrate the XPS spectra. After the calibration, the corresponding narrow-range W 4f, S 2p, and O 1s spectra were discussed in detail. Figure 5b shows that the O 1s spectra of the WS_2_/WO_3_ heterostructures can be well fitted into two peaks at 530.94 eV and 531.94 eV, attributed to the O in WO_3_ and chemisorbed oxygen-containing materials, respectively [31,32]. As shown in Figure 5c, the two peaks of W 4f5/2 and W 4f7/2 of WS_2_ are situated at about 34.60 eV and 33.24 eV. After partial oxidation, new peaks at 37.99 eV and 35.96 eV appeared, which could be assigned to W 4f5/2 and W 4f7/2 in WO_3_ [33]. The characteristic peaks of both WS_2_ and WO_3_ can be clearly observed in WSO-n, which further confirms the successful synthesis of the aimed heterostructures. Ultimately, the S 2p spectra of these composites were characterized (Figure 5d). There are two binding energy of S 2p located at 162.30 eV and 163.70 eV, which can be assigned to the presence of S^2−^ in WS_2_. The special peak present at 169.10 eV could be attributed to S^6+^ due to the oxidation of divalent sulfide ions [34]. Significantly, it only existed in WS_2_/WO_3_ hybrids. The characteristic peaks of S decreased gradually with the oxidation process due to the enhanced transformation from WS_2_ to WO_3_. Overall, all the XPS spectra results mentioned above demonstrated a successful construction of WS_2_/WO_3_ heterostructures.

**Figure 6:**
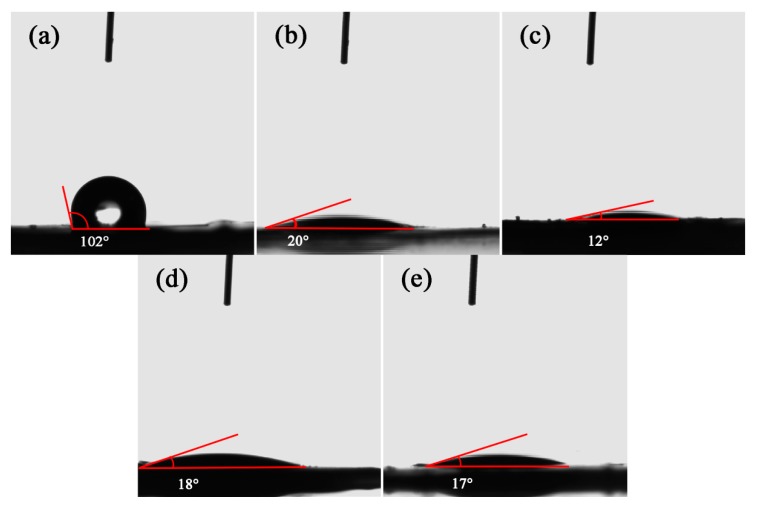
Water Contact angles of (**a**) WS_2_, (**b**) WSO-5, (**c**) WSO-30, (**d**) WSO-60, and (**e**) WO_3_.

A contact angle analysis was adopted to assess the obtained materials’ wettability, which is traditionally determined by the substrates’ morphologies and chemical compositions [35,36]. Initially, the contact angle of pure WS_2_ was measured to be about 102° (Figure 6a). After oxidization, a sharp decrement of the contact angle values was observed with all values lower than 20° (Figure 6b–e), indicating the hydrophilicity of WS_2_/WO_3_ hybrids, and WO_3_ was dramatically promoted with the introduction of oxygen atoms. The increasing wettability might result from the formation of hydrogen bonds which are introduced by strong-polar oxygen atoms interacting with water molecules [37,38]. In addition, the high–magnification SEM images of WS_2_/WO_3_ hybrids (Figure 2c–e) shows that these heterostructures have rougher surface morphologies with voids and many highly water-permeable channels, which can further enhance the hydrophilicity.

### 3.2. Adsorption Knetics

**Figure 7:**
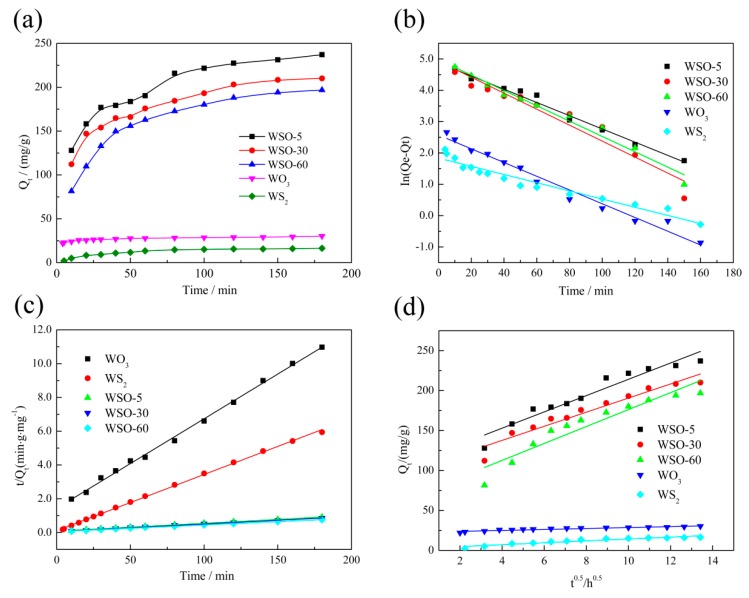
(**a**) The effect of the contact time on the adsorption capacity of WS_2_, WS_2_/WO_3_, and WO_3_ for RhB; the fitted kinetic data with (**b**) the pseudo-first-order model, (**c**) pseudo-second-order model, and (**d**) Weber-Morris model.

To explore the adsorption kinetics of these newly developed materials on RhB, the effect of the adsorption capacity over contact time was evaluated. As shown in Figure 7a, all the WS_2_/WO_3_ hybrid samples exhibited similar trends, with the sorption capacities increasing rapidly during the first 100 min, then gradually reaching equilibrium with a further increase of the adsorption time. Obviously, the pure WS_2_ showed a poor adsorption capacity of merely 16.4 mg/g, which can be ascribed to the hydrophobic surface property. After the introduction of oxygen atoms with a short duration time of 5 min, the adsorption capacity got a tremendous promotion (237.1 mg/g) due to the stronger hydrophilicity, as well as to the retention of the superior properties of WS_2_. However, with the further increment of the oxidation duration, the adsorption capacity gradually declined until reaching pure WO_3_, which exhibited a low adsorption performance of 30.1 mg/g. 

Three empirical kinetic models were adopted to conduct a further analysis toward the adsorption kinetics, namely, a pseudo-first-order model (Equation (3)), pseudo-second order model (Equation (4)), and Weber-Morris model (Equation (5)):

(3) $$\ln\left(Q_{e}-Q_{t}\right)=\ln Q_{e}-k_{1}t,$$

(4) $$\frac{t}{Q_{t}}=\frac{1}{k_{2}Q_{e}^{2}}+\frac{t}{Q_{e}},$$

(5) $$Q_{t}=k_{i}t^{1/2}+c,$$

where $Q_{e}$ (mg/g) and $Q_{t}$ (mg/g) represent the adsorption capacities of RhB onto samples at equilibrium and time t (min); c (mg/g) is a constant; $k_{1}$(min^−1^), $k_{2}$ (g·mg^−1^·min^−1^), and $k_{i}$ (mg·g^−1^·min^−1/2^) are the rate constants of the pseudo-first order, pseudo-second order, and Weber-Morris models, respectively. 

The fitting results are displayed in Figure 7b–d, and the calculated kinetic parameters are summarized in Table S1. Compared with the pseudo-first-order model and Weber-Morris model, the pseudo-second-order model exhibits better correlation results for the experimental data, with a higher *R*^2^ value (above 0.99). 

### 3.3. Adsorption Isotherms

**Figure 8:**
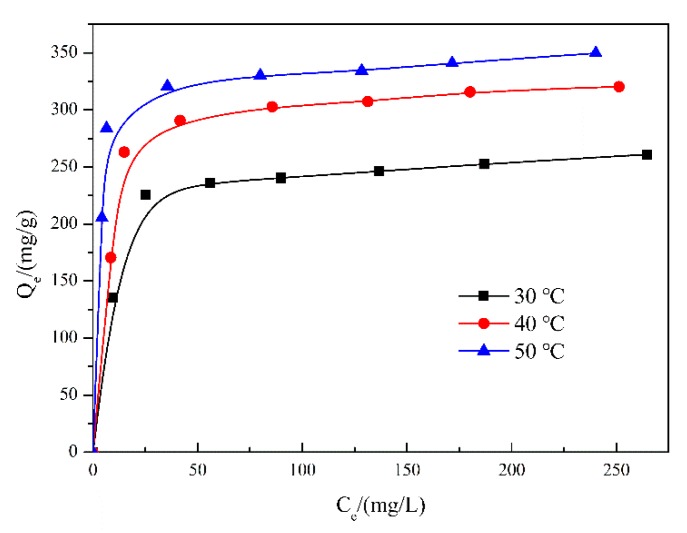
The adsorption isotherms of RhB on WSO-5 at pH 6. (30, 40 and 50 °C).

To elucidate the equilibrium states of RhB dye molecules on materials and in liquid surroundings, the adsorption isotherms were conducted [39]. In particular, WSO-5 was chosen as the model material due to its higher adsorption capacity. The adsorption isotherms of RhB on WSO-5 were carried out at gradient temperatures of 30, 40 and 50 °C. As shown in Figure 8, the adsorption isotherms exhibited conventional L-types with obvious platforms. The results showed that the increasing temperature in circumstance could facilitate the adsorption for WSO-5, suggesting an endothermic adsorption process. Furthermore, a straightforward statistical physics model was utilized to fit the adsorption isotherms of RhB. The mathematical equation of the model is presented as follows [40]:

(6) $$Q_{e}=\frac{nD_{m}}{1+{\left(\frac{c_{1/2}}{c_{e}}\right)}^{n}},$$

where n represents the number of RhB molecules per site of adsorbent, $D_{m}$ (mg/g) is the density of the receptor sites, and $c_{1/2}$ (mg/L) means the concentration at half saturation of the formed layer. According to the statistic physics model, the adsorption capacity at saturation $Q_{sat}$ is given by [41]:

(7) $$Q_{sat}=nD_{m}.$$

The expression of the adsorption energy E, which contains the solubility $c_{s}$ (mg/L) of the adsorbates, can be expressed as follows [41,42]:

(8) $$E\;=RTln(c_{s}/c_{1/2}).$$

The fitted model parameters were determined through nonlinear regressions of the experimental sorption isotherms (Table 1). The values of $R^{2}$ are close to unity, indicating that this monolayer model could be used to elucidate the sorption mechanisms of the test adsorbents.

**Figure 9:**
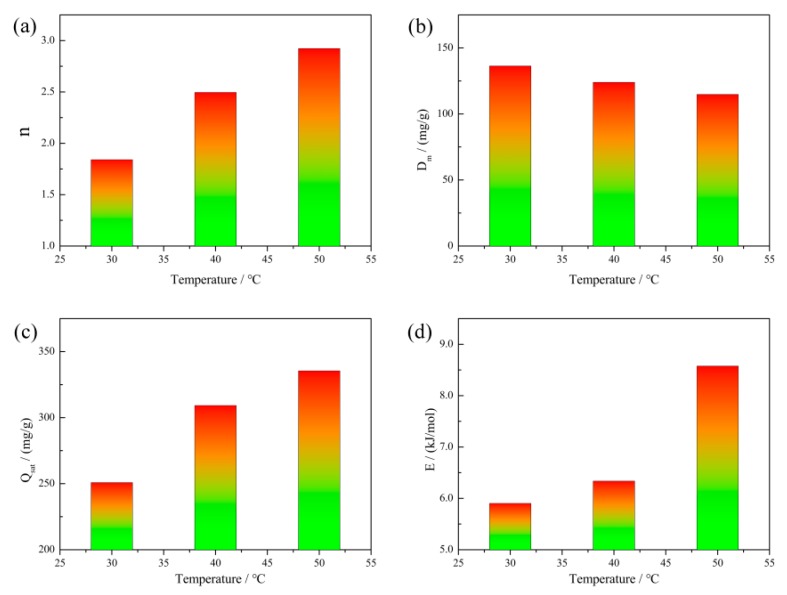
(**a**) The effect of the temperature on the number of molecules per site, (**b**) the density of receptor sites, (**c**) the adsorption capacity at saturation, and (**d**) the adsorption energy of WSO-5.

The parameter n gives information about the orientation states of the adsorbates on the adsorbent surface. Traditionally, two situations are taken into consideration: if n<1, the adsorbates would interact with the multifunctional groups of adsorbents (horizontal orientation); if n≥1, more than one adsorbate molecules would be anchored in one receptor site on the adsorbent surface (non-horizontal orientation) [43]. The effects of the temperature on this parameter, displayed in Figure 9a, show that the values of n in all tested temperature are above unity, varying from 1.84 to 2.92. In consequence, this evidence implies a multi-molecular adsorption process of RhB molecules with an oblique orientation on WS_2_/WO_3_ [40].

Moreover, the effect of the temperature on the receptor sites’ density $D_{m}$ was also interpreted, and the results are shown in Figure 9b. This decreasing tendency is related to the increasing number of molecules captured per site, leading to a tendency of aggregation which would hinder the accessibility of the RhB molecules to the adsorption receptor sites [42,44].

The correlation of the parameters (n and $D_{m}$) of the monolayer can be examined by the variation of the adsorption capacity on the temperature. As shown in Figure 9c, the effect of the temperature on the parameter $Q_{sat}$ indicates that the increment of temperature results in an increasing $Q_{sat}$ value, which further verifies an endothermic process of RhB adsorption.

The adsorption energy E was calculated to further clarify the interactions between the RhB molecules and the adsorbent surface. The E values were calculated via Equation (8), and the results that E evolve with the tested temperature are displayed in Figure 9d. The results confirm again that the adsorption is an endothermic process, which matches the analysis conclusion of $Q_{sat}$ well. Furthermore, the results show that the adsorption is a physisorption process within the investigated temperatures, since all E values are lower than 10 kJ/mol [42].

### 3.4. Effect of Adsorbent Dosage and Solution pH

#### 3.4.1. Effect of Adsorbent Dosage

**Figure 10:**
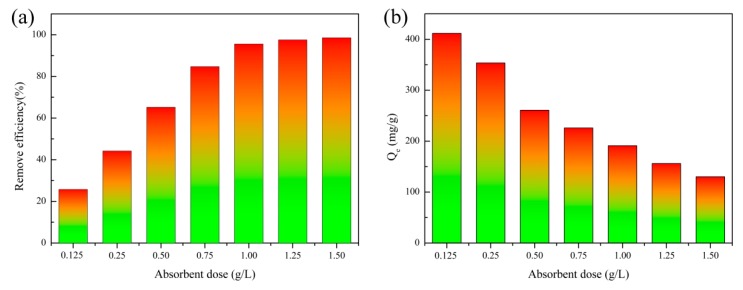
(**a**) The adsorption percentage and (**b**) adsorption amount of RhB on WS_2_/WO_3_ as a function of the adsorbent content at 30 °C, pH 6.0, and $C_{\mathrm{initial}}$ 200 mg/L.

The cost of an adsorbent in practical application depends, to a large extent, on its specific dosage to achieve the wastewater treatment standards. In this work, the correlation of the adsorbent dosage and the removal efficiency of RhB was explored (Figure 10). It can be clearly observed in Figure 10a that the removal efficiency of RhB increases from about 24% to almost 95% as the dosage increases from 0.125 to 1.5 g/L. However, there is no evident growth anymore when the dosage exceeds 1.0 g/L. The reason for this might be that more available binding sites are provided to capture RhB molecules in solution with the increased content of WS_2_/WO_3_ composites. To reduce the expense of sewage disposal, an optimal WS_2_/WO_3_ dosage of 1 g/L is suggested for the efficient elimination of RhB, with an initial concentration of 200 mg/L at pH 6.0.

Meanwhile, the mass-based adsorption amount of RhB dye gradually decreased with the increment of solid content (Figure 10b). This can be explained by the fact that WS_2_/WO_3_ microflowers dispersed well in solution and that the surface active sites were highly available to form a stable complex with RhB molecules at low dosages. However, higher solid dosages would cause the collision and aggregation of the WS_2_/WO_3_ adsorbent, resulting in a decrease in the density of the active sites. In addition, under a vigorous stirring rate (200 rpm), the inter-collision between WS2/WO3 at a higher solid content would also cause the desorption of some weak-linked RhB molecules and ultimately reduce the adsorption amount [22,45].

#### 3.4.2. Effect of Solution pH

**Figure 11:**
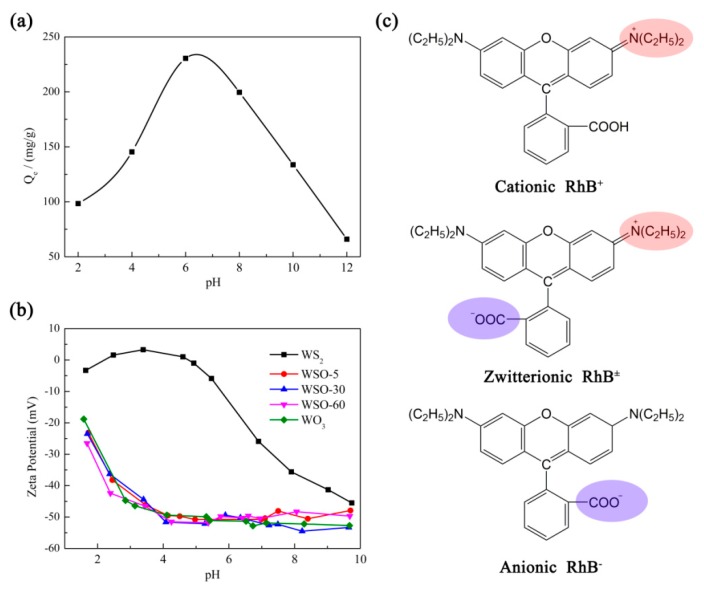
(**a**) The effect of the pH on the adsorption of RhB on WSO-5. (Adsorbent = 20 mg, RhB = 40 mL of 200 mg/L, *T* = 30 °C.) (**b**) The Zeta potential of WS_2_, WS_2_/WO_3_ hybrids, and WO_3_ at various pH values. (**c**) The molecular structures of RhB in its zwitterionic, cationic, and anionic form, respectively.

Usually, the solution pH is a significant factor that exerts great influence on the adsorption of dyes onto the adsorbents. Therefore, the effect of the pH on RhB adsorption for the WS_2_/WO_3_ hybrid was investigated, and the results are exhibited in Figure 11a. The equilibrium adsorption amount $Q_{e}$ (mg/g) exhibited an increasing trend when raising pH values from 2 to 6 and then sharply decreased when the initial pH values exceeded 7. This trend suggests that WS_2_/WO_3_ exhibits an excellent performance for RhB adsorption under neutral or slightly acidic circumstances, which can be interpreted by the correspondingly extraordinary negative Zeta potentials of the WS_2_/WO_3_ materials displayed in this pH range (pH ≥ 4) (Figure 11b) that facilitate the attraction of RhB dye molecules.

The interrelation of the existing forms of RhB in solution and the adsorption capacity were further studied. It has been reported that RhB molecules exist in three forms in solution, including the cationic, anionic, and zwitterionic forms (Figure 11c) [46]. In an acidic environment, the RhB molecule is ionized and exists predominantly in its cationic form (RhB^+^). However, the competitive protons introduced by the acidic environment exert a positive charge effect on the WS_2_/WO_3_ surface, which is reflected by the Zeta potential analysis (Figure 11b). Therefore, under these circumstances it is difficult for WS_2_/WO_3_ to come into contact with RhB^+^ molecules, owing to the electrostatic repulsion. With gradually increased pH values, the fraction of the cationic form (RhB^+^) decreased, while the zwitterionic form (RhB^±^) increased simultaneously. These zwitterionic RhB molecules, with both C=N^+^ and COO^−^ groups, would aggregate on the active sites, resulting in a higher adsorption capacity [47,48]. When the pH rose up to a higher value above 7, the anionic form (RhB^−^) increased, and the adsorption capacity of WS_2_/WO_3_ significantly decreased owing to the electrostatic repulsion between negatively charged RhB^-^ and negative WS_2_/WO_3_.

### 3.5. Adsorption Mechanism

**Figure 12:**
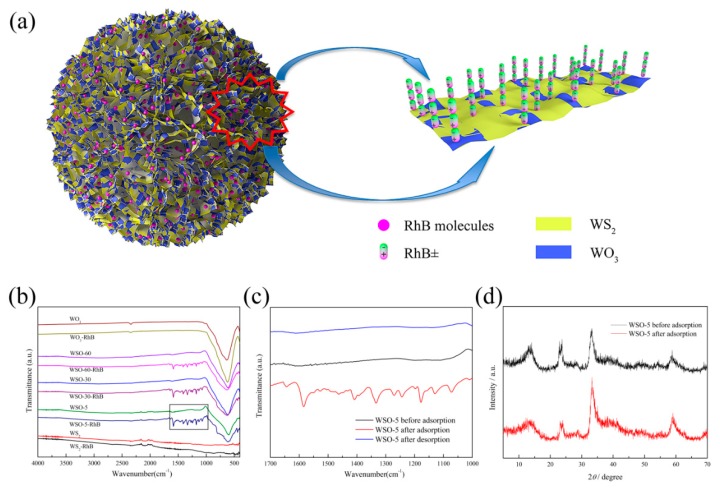
(**a**) The adsorption mechanism of WSO-5 microflowers toward RhB; (**b**) the FTIR spectra of WS_2_, WS_2_/WO_3_, and WO_3_ before and after the adsorption of RhB dye; (**c**) The enlarged image of WSO-5 of the zone marked in (**b**); and (**d**) the XRD patterns of WSO-5 before and after the adsorption of RhB dye.

The adsorption mechanism of RhB dye onto WS_2_/WO_3_ heterostructures was further elucidated by considering all the results discussed above. As shown in Figure 12a, the electronegative sulfur and oxygen atoms in the polar active sites of the WS_2_/WO_3_ heterostructures would connect with the RhB^±^ molecules by electrostatic attraction, subsequently inducing the adsorption process. According to the fitting results of the monolayer model mentioned before, RhB^±^ molecules exhibit a non-horizontal orientation on the WS_2_/WO_3_ surface. Specifically, this is primarily conducted via the RhB^±^ molecules aggregating layer-by-layer on the active sites in a head-to-tail way, since this zwitterionic form has positive and negative sites simultaneously [22]. The adsorption of RhB molecules onto materials can be detected through a comparison of the FTIR spectroscopy before and after adsorption toward RhB (Figure 12b). The peak at about 580 cm^−1^ in the spectra of WS_2_/WO_3_ is assigned to O–W–O vibration. Meanwhile, the new peaks at about 1590 and 1340 cm^−1^ belonging to the characteristic peaks of in-ring C–C and C–H stretching vibrations in the aromatic ring of the RhB molecule could be detected after the adsorption of dye (Figure 12c) [26,49]. However, there are no obvious dye characteristic peaks in the spectra of WS_2_ and WO_3_, which indicates that few RhB molecules are adsorbed onto these two materials. Besides, no characteristic peaks of RhB were observed after the desorption process, which means that electrostatic attraction is responsible for the RhB adsorption by WS_2_/WO_3_. On the other hand, the XRD patterns in Figure 12d also prove that the adsorption process didn’t change the crystal structure of WSO-5. The characteristic peaks are well retained after adsorption, and there are no obvious peaks shifts, which demonstrates that the adsorption is a complete physical behavior that occurs primarily through electrostatic attraction.

### 3.6. Recyclability

**Figure 13:**
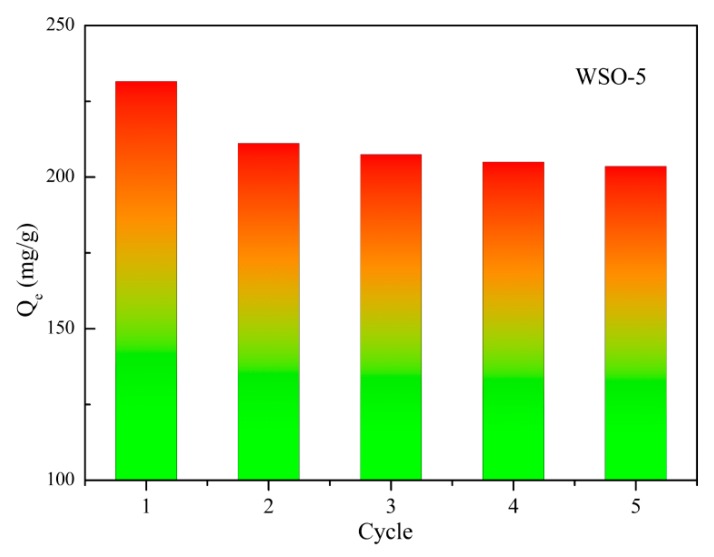
The recyclability of WSO-5.

For the practical application of an adsorbent, a quick adsorption rate and high adsorption capacity are of great significance, and the reusability of the adsorbent should be taken into consideration as well. After the adsorption experiments, the adsorbent was washed with ethanol as the eluting agent and reused for the next adsorption process. According to Figure 13, the adsorption performance of WSO-5 toward RhB dye slightly decreased after the first cycle, while remaining almost constant in the following cycles. These results demonstrate that WS_2_/WO_3_ has a good stability and can be regenerated and reused efficiently for the treatment of waste water contaminated with RhB dye.

### 3.7. Comparison with Other Adsorption Materials

To sum up, in this study the WS_2_/WO_3_ hybrids were first synthesized and utilized to investigate their dye adsorption performance. The comparison of the mass-based adsorption performance between them and some other reported TMD absorbents is presented in Table 2. It can be clearly seen that the WS_2_/WO_3_ hybrids, particularly WSO-5, exhibit a higher adsorption capacity and relatively medium adsorption time. Because of the multiple advantages, such as a simple synthesis process, high removal efficiency, and good reusability, the WS_2_/WO_3_ heterostructure could be a promising candidate for the elimination and separation of organic dyes from wastewater.

## 4. Discussion

In this work, a new type of WS_2_/WO_3_ heterostructure, which could be used as excellent absorbents, was developed and fabricated by coupling a solvothermal synthesis method with a partial oxidation strategy. Different characterization results confirmed the successful fabrication of WS_2_/WO_3_ heterostructures, which possess more negative surface charges and which highly promoted hydrophilicity after a partial oxidation. The synthesized heterostructures were employed for the decontamination of dyes, and the results indicate that WSO-5 has an enhanced adsorption capacity of 237.1 mg/g, compared with pure WS_2_ and WO_3_, which exhibited a limited capacity of 16.4 and 30.4 mg/g, respectively. Besides, three models were applied to simulate the kinetics in the adsorption process, and the results prove that the pseudo-second-order model gives better fitting results. Furthermore, the adsorption isotherms were also conducted by a precise monolayer adsorption model to reveal the interaction between the adsorbent and adsorbate, demonstrating a non-horizontal orientation of the RhB molecules and an endothermic physisorption process. Finally, the recyclability study showed that WS_2_/WO_3_ can be easily regenerated through a simple ethanol washing and that the adsorption performance was well-maintained after five cycles. These results indicate that WS_2_/WO_3_ can be a promising adsorbent for the removal of RhB dye from wastewater. Overall, the partial oxidation strategy can introduce more negative surface charges and enhance hydrophilicity, which gives an indication for the surficial modification of other TMD materials and their application in the elimination of dyes.

## Figures and Tables

**Table 1:** The parameters of the monolayer adsorption model under different tested temperatures.

T (°C)	n	$D_{m}$ (mg/g)	$Q_{sat}$ (mg/g)	E (kJ/mol)	$R^{2}$
30	1.840	136.3	250.8	5.901	0.9993
40	2.495	123.9	309.1	6.336	0.9992
50	2.922	114.8	335.4	8.575	0.9993

**Table 2:** A comparison of the adsorption performance of the WS_2_, WO_3_, and WS_2_/WO_3_ heterostructures with other reported TMD adsorbents.

Adsorbents	Dyes	Adsorption Capacity (mg/g)	Adsorption Time (min)	References
MoS_2_ nanosheets	RhB	163	420	[27]
2D MoS_2_ nanosheets	MB	146	5	[50]
Flower-like MoS_2_	RhB	55.0	180	[51]
MoS_2_-glue sponges	RhB	127	60	[22]
Fe_3_O_4_/MoS_2_	RhB	22.0	30	[52]
Flower-like WS_2_	RhB	16.4	100	This work
WO_3_ particles	RhB	30.4	100	This work
Flower-like WSO-5	RhB	237	100	This work

## Supplementary Materials

The following are available online at https://www.mdpi.com/2079-4991/10/2/278/s1, Figure S1: TG curve of the as-prepared WS_2_; Table S1: Kinetic parameters of the WSO-n, WS_2_, WO_3_ samples.
