# Supplementary material for: Partial Oxidation Strategy to Synthesize WS2/WO3 Heterostructure with Enhanced Adsorption Performance for Organic Dyes: Synthesis, Modelling, and Mechanism
Source: Nanomaterials (Basel). 2020 Feb 6;10(2):278. doi: 10.3390/nano10020278 (PMC7075182; doi:10.3390/nano10020278)
Supplement: Supplementary file 1 [file nanomaterials-10-00278-s001.pdf]

Supporting Information for

Partial Oxidation Strategy to Synthesize WS_2/WO_3 Heterostructure with Enhanced Adsorption Performance for Organic Dyes: Synthesis, Modelling, and Mechanism

Guiping Li [1], Yongli Wang [1,2,*], Jingtao Bi [1], Xin Huang [1,2], Yafei Mao [1], Liang Luo [1] and Hongxun Hao [1,2,*]

[1] National Engineering Research Center for Industry Crystallization Technology, School of Chemical Engineering and Technology, Tianjin University, Tianjin 300072, China; ligp@tju.edu.cn (G.L.); jingtaob@gmail.com (J.B.); x_huang@tju.edu.cn (X.H.); maoyafei@tju.edu.cn (Y.M.); 2016207086@tju.edu.cn (L.L.)

[2] Co-Innovation Center of Chemical Science and Engineering, Tianjin 300072, China

* Correspondence: yliwang@tju.edu.cn (Y.W.); hongxunhao@tju.edu.cn (H.H.)

Electronic Supplementary Information (ESI) available.

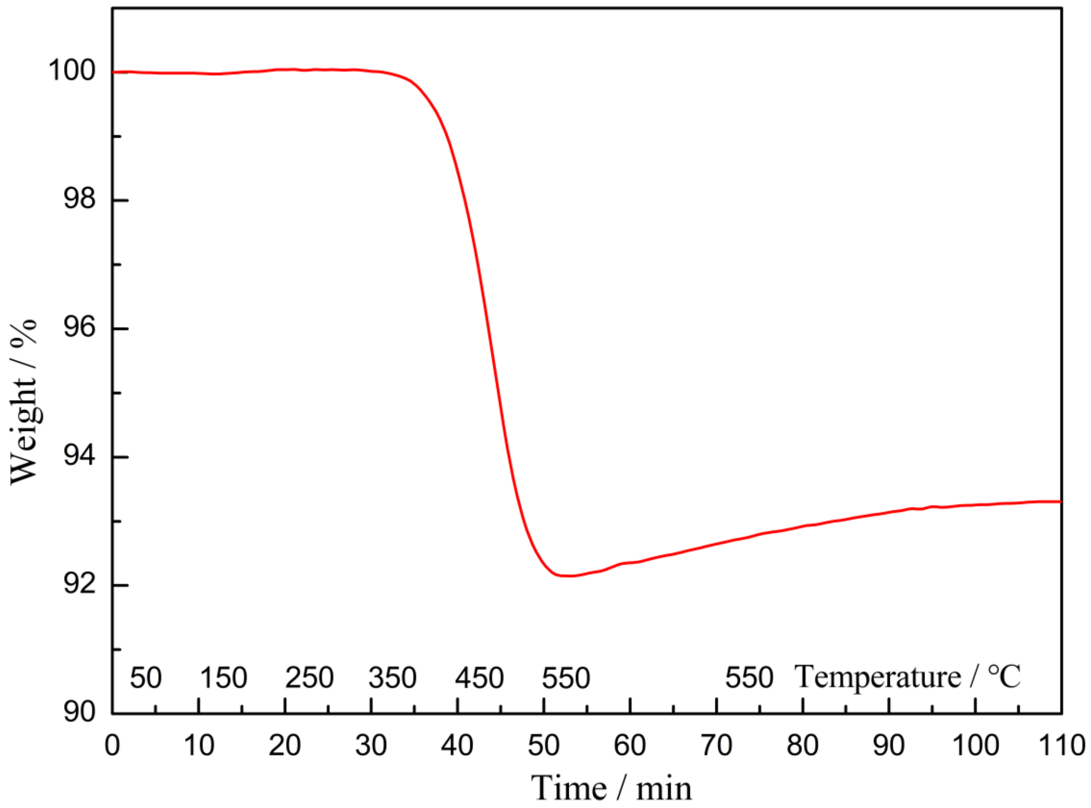

Figure 1. TG curve of the as-prepared WS_2. Note: The heating program was set at ramp rate of 10 K/min. When the heating temperature reached 550 ℃, the program entered into thernmostatically control. The herizotal axis has two means of expression, one is tepemrerature (above) and another is time (below).

Table S1. Kinetic parameters of the WSO-n, WS_2, WO_3 samples

Samples	$Q_{e,exp}$ (mg/g)	Pesudo-first-order model		
		K_1(1/min)	$Q_{e,cal}$ (mg/g)	R^2
WSO-5	237.1	0.0211	259.4	0.982
WSO-30	210.2	0.0255	250.2	0.932
WSO-60	196.9	0.0244	225.6	0.975
WO_3	30.3	0.0219	35.1	0.976
WS_2	16.4	0.0131	11.3	0.940
Samples	$Q_{e,exp}$ (mg/g)	Pesudo-Second-order model		
		K_1 (1/min)	$Q_{e,cal}$ (mg/g)	R^2
WSO-5	237.1	2.71×10^{-4}	253.8	0.9967
WSO-30	210.2	3.18×10^{-4}	224.7	0.9973
WSO-60	196.9	2.44×10^{-4}	216.9	0.9995
WO_3	30.3	9.49×10^{-3}	30.1	0.9990
WS_2	16.4	1.94×10^{-3}	18.9	0.9975
Samples	$Q_{e,exp}$ (mg/g)	Weber-Morris model		
		K_i/(mg·g·min$^{-1/2}$)	c/(mg/g)	R^2
WSO-5	237.1	10.20	112.2	0.9289
WSO-30	210.2	8.834	102.4	0.9266
WSO-60	196.9	10.60	70.30	0.8899
WO_3	30.3	0.5999	22.56	0.8933
WS_2	16.4	1.182	2.389	0.8845

(Note: $Q_{e,cal}$ is the fitted adsorption capacities; $Q_{e,exp}$ is the experimental equilibrium values at 3 h.)
